# Monitoring the Effect of Metal Ions on the Mobility of *Artemia salina* Nauplii

**DOI:** 10.3390/bios1020036

**Published:** 2011-03-28

**Authors:** Varvara Kokkali, Ioannis Katramados, Jeffrey D. Newman

**Affiliations:** 1 Cranfield Health, Vincent Building, Cranfield University, Cranfield, Bedfordshire, MK43 0AL, UK; E-Mail: v.kokkali.s06@cranfield.ac.uk; 2 School of Engineering, Whittle building, Cranfield University, Cranfield, Bedfordshire, MK43 0AL, UK; E-Mail: i.katramados@cranfield.ac.uk

**Keywords:** *Artemia salina*, crustaceans, image processing, mobility, toxicity assay

## Abstract

This study aims to measure the effect of toxic aqueous solutions of metals on the mobility of *Artemia salina* nauplii by using digital image processing. The instrument consists of a camera with a macro lens, a dark chamber, a light source and a laptop computer. Four nauplii were inserted into a macro cuvette, which contained copper, cadmium, iron and zinc ions at various concentrations. The nauplii were then filmed inside the dark chamber for two minutes and the video sequence was processed by a motion tracking algorithm that estimated their mobility. The results obtained by this system were compared to the mortality assay of the *Artemia salina* nauplii. Despite the small number of tested organisms, this system demonstrates great sensitivity in quantifying the mobility of the nauplii, which leads to significantly lower EC_50_ values than those of the mortality assay. Furthermore, concentrations of parts per trillion of toxic compounds could be detected for some of the metals. The main novelty of this instrument relies in the sub-pixel accuracy of the tracking algorithm that enables robust measurement of the deterioration of the mobility of *Artemia salina* even at very low concentrations of toxic metals.

## 1. Introduction

The ongoing interest in assessing the environmental effect of toxic waste materials has led to an increasing number of bioassays and screening devices. Such techniques are often based on measuring the effect of toxic substances on the behaviour and life cycle of microorganisms, invertebrates and algae. Different biological properties that have been utilized for toxicity screening include the luminescence of *Vibrio fischeri*, respiratory rate of aerobic bacteria, enzyme activity of *Daphnia magna*, fluorescence of *Chlorella vulgari (algae*) and the lethality to crustaceans (such as *Artemia salina*). In addition, several studies have specifically focused on the impact of metals to the biota, by analysing their toxic effect to crustaceans. Giarratano *et al*. [1] and Lorenzon *et al*. [2] have investigated the effect of heavy metals to *Exosphaeroma gigas* and *Palaemon elegans* respectively. Furthermore, MacRae and Pandey [3] have researched the relation between water toxicity and hatching success of *Artemia* species. Similarly, Gajbhiye and Hirota [4] have proved that the lethality of these species is dependent on the concentration of heavy metals in water.

*Artemia* species, or brine shrimps, have also been used in many scientific experiments for acute toxicity testing of toxic materials including pesticides (Barahona & Sánchez-Fortún [5]), leachates (Svensson *et al*. [6]), dental materials (Pelka *et al*. [7]), fungal toxins (Harwig & Scott, [8]) and antifouling biocides (Koutsaftis and Aoyama [9]). Although *Artemia* species are not considered as sensitive as other screening instruments or organisms (Nunes *et al*. [10]), they have some important advantages including constant commercial availability all year round, cost efficiency, ease of culture, short life-cycle, no feeding required during the assay and great offspring production (Vanhaecke *et al*. [11]).

These advantages have led to a wide range of *Artemia*-based bioassays. The determination of the LC_50_ of the nauplii (instar II-III stage) (Vanhaecke and Persoone [12]), the hatchability of the cysts (MacRae and Pandey [3]), the different age specimens (Barahona and Sánchez-Fortún, [5]) and the disruptions on an enzyme property (Varó *et al*. [13,14]) are only some of the *Artemia* end-points that have already been examined as evidence of toxicity. Specifically, Vanhaecke and Persoone [12] have demonstrated a shrimp-based bioassay, known as the ARC-test (*Artemia* Reference Centre), with highly reproducible and accurate results. Additionally, Vanhaecke *et al*. [15] proved that the most sensitive age for the majority of the tested compounds were the 48-h old nauplii at the stage of instar II-III. The same results have also been supported by Barahona and Sánchez-Fortún [5] and Togulga [16]. On the other hand, *Artemia* species are very resistant to metals and accumulate them without any obvious effect on their life-cycle (Sarabia *et al*. [13]). 

This study is based on a patented device by Portmann *et al*. [17], which has been redesigned to be more accurate in recording small changes in the mobility of *Artemia salina* nauplii using a monocular camera and a digital image-processing algorithm. The main hypothesis of this work was that the accumulation of toxic compounds to *Artemia* organism has a deteriorating effect on its mobility and this deterioration can be measured. Potassium dichromate was firstly tested as a reference substance since it is a widely applied reference toxic compound in aquatic toxicology (Vanhaecke *et al*. [15]). Later, copper, cadmium, iron and zinc salts were tested for their lethal effect on *Artemia salina* nauplii. Furthermore, the survivors were also counted and their hypothetical mobility was estimated. Finally, the mobility of the nauplii in those toxic metal samples was recorded and compared to the hypothetical one. The results showed that concentrations of parts per trillion of toxic metals affected the mobility of the nauplii and these changes could be detected consistently using the proposed technique. 

## 2. Materials and Methods

### 2.1. Preparation of the Test Organisms

*Artemia* cysts (San Francisco Strain Brine shrimps) were incubated in artificial seawater (ASW) of 3.5% salinity, at 30 °C, pH 8.0 ± 0.5 and under constant aeration for 48 h until they reach the stage of instar II-III. The temperature of the samples throughout the experiment remained at 25 °C. Nauplii from the same generation were applied for each compound.

### 2.2. Test Chemicals

The reagents were of general laboratory grade unless stated otherwise. Potassium dichromate was provided from BDH (Lutterworth, UK). Copper sulphate [CuSO_4_·5H_2_O] and cadmium chloride [CdCl_2_·H_2_O] were obtained from Sigma-Aldrich (Gillingham, UK). Copper nitrate [Cu (NO_3_)_2_·3H_2_O] was provided from Fisher Scientific (Loughborough, UK). Zinc sulphate [ZnSO_4_·7H_2_O] and iron sulphate [FeSO_4_·7H_2_O] were obtained from Acros Organics (Loughborough, UK). Stock solutions of 10,000 mg/L of all reagents were prepared in distilled water and then serial dilutions in ASW were prepared for each metal. The pH of the samples was 8.0 ± 0.5. Macro-cuvettes from polystyrene were used as containers for the toxic samples provided by Fischer Scientific. The same samples of each toxic compound were applied for the assessment of the toxicity using the mortality based assay, hypothetical mobility and mobility assay.

### 2.3. Further Equipment

A monocular camera (Canon Power shot S3 IS) and a laptop were used to track the movement of the nauplii by recording their activity for two minutes and processing the videos. Light-emitting diodes (LED) torches (AAA battery torch, 7dayshop, Guernsey) were used as illumination source. Subsequently, the captured video sequence was analysed by a digital image processing algorithm that estimates the mobility of the nauplii. The concept has been based on the U.S. Patent of Portmann *et al*. [17]. 

### 2.4. Mortality-Based Assay

The *Artemia* cysts were cultured in ASW until the stages of instar II-III nauplii (48 h old). Four mL of either ASW (controls) or the tested compound was added to each cell. Then, four nauplii were transferred with Pasteur pipette with the minimal of ASW carried over. Five replicates were performed for the controls and four for every toxicant dilution. After 24 h of incubation, the dead nauplii were counted and the LC_50_ values estimated with probit analysis. The assay would be considered valid if the mortality percentage of the control does not exceed the 10% (Vanhaecke *et al*. [11]) and the estimated LC_50_ of the reference substance (potassium dichromate) ranges between 30 and 50 mg/L (Svensson *et al*. [6]).

### 2.5. Hypothetical Mobility of the Nauplii

**Figure 1:**
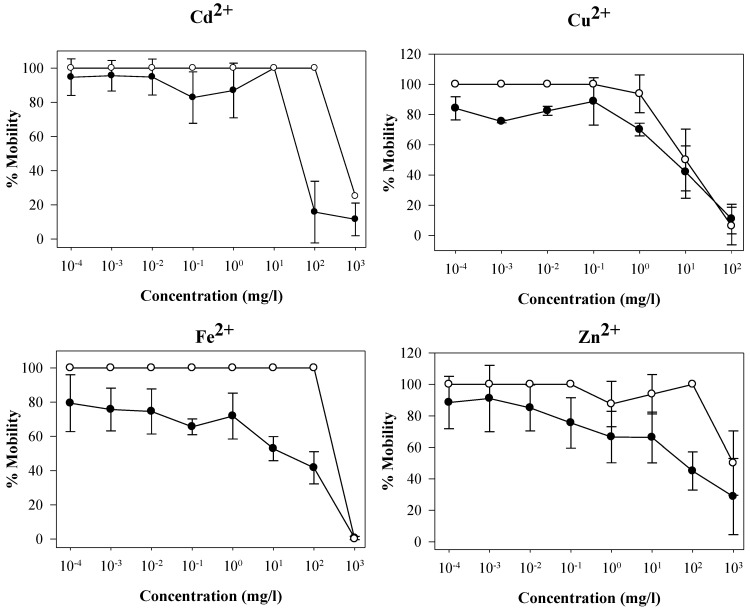
Percentage of (o) hypothetical and (●) measured mobility (using the camera-based instrument) of the *Artemia salina* nauplii for Cadmium, Iron, Copper and Zinc ions. The error bars represent the standard error from the four replicates of each concentration.

The main advantage of the proposed approach is that it can quantitatively measure subtle changes in the mobility of nauplii and as a result detect small concentrations of toxic substances. Two minute-videos of five replicates of four nauplii in ASW were recorded and processed through the digital image processing algorithm. In order to calculate the “% hypothetical mobility” of a specific sample, the following equation was applied (Equation (1)). 


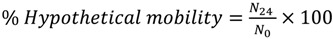
(1)
where *N_24_* is the number of the nauplii which remained living after 24 h of exposure to the toxic substance and *N_0_* is the average number of the nauplii initially inserted into the control sample. This approach essentially makes the assumption that alive nauplii have retained 100% of their initial mobility after 24 h in the toxic solutions. In Figure 1, the hypothetical mobility is plotted against the actual mobility of the nauplii. The corresponding “best fit model” equations are displayed on Table 1.

**Table 1:** Equations from the curves of hypothetical and measured mobility using the “best fit model”.

Compound	Equation and R^2^ from %mobility graphs	Equation and R^2^ from %hypothetical mobility graphs
Cadmium chloride	y = 0.0008x^2^ − 0.8406x + 93.713	y = −0.00008x^2^ + 0.0084x + 99.987
	R^2^ = 0.9606	R^2^ = 1
Copper sulphate	y = 0.0366x^2^ − 4.3644x + 81.278	y = 0.0452x^2^ − 5.4572x + 99.97
	R^2^ = 0.9683	R^2^ = 0.9999
Iron sulphate	y = 0.0003x^2^ − 0.3341x + 70.83	y = −0.0001x^2^ + 0.0112x + 99.983
	R^2^ = 0.9267	R^2^ = 1
Zinc sulphate	y = 0.0003x^2^ − 0.3933x + 79.705	y = −0.00008x^2^ + 0.0344x + 96.893
	R^2^ = 0.8493	R^2^ = 0.9336

*NE: Not Estimated.

### 2.6. Mobility Assay Using Digital Image Processing

**Figure 2:**
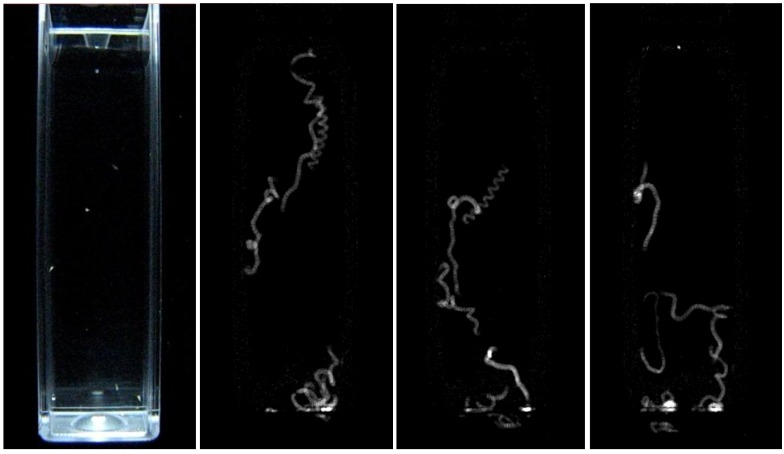
The left image shows a representative video frame as captured by the camera. A sequence of such frames forms the input to the digital image processing algorithm. Although the shrimps are not clearly visible in the cuvette, they can be tracked with good accuracy in order to measure their mobility. The remaining images show some characteristic examples of tracking paths which were derived over 6.6 s intervals. Lighter gray shades denote areas of higher mobility including overlapping tracking paths

Video sequences were recorded for two minutes for all the controls and samples. All the cells were properly illuminated with LED torches. In each video sequence, the nauplii were counted and tracked as illustrated in Figure 2. The % mobility of the nauplii in each sample was estimated according to the following Equation (2):

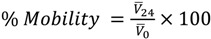
(2)
where *V_24_* is the average speed per nauplius in a specific sample after 24 h of exposure to the toxicant as was estimated automatically from the algorithm and *V_0_* is the average speed per nauplius in the control samples.

The Effective Concentration (EC_50_) was estimated through the “best fit model” (Excel, Microsoft Office, 2007). The best fit model revealed that the most appropriate equation was the quadratic equation (Table 1).

### 2.7. Statistical Analysis

Statistical analysis of one-way ANOVA of the data was performed using Genstat v.12.0 (VSN International Ltd, Hemel Hempstead, UK) and was evaluated at the p < 0.001 level, as presented in Table 2. Furthermore, the 24 h LC_50_ was determined by using Biostat v.5.2.5.0 (AnalystSoft, 2008). Finally, two-sample t-test was performed for the results obtained from the hypothetical and actual mobility of the nauplii and they were evaluated for their significance at the p < 0.05 level using SigmaPlot v.11.0 (Systat Software, 2008).

**Table 2:** One-way ANOVA table of the mobility of the nauplii monitored in Cadmium, Iron, Copper and Zinc solutions.

Substance	Source of variation	Degrees of freedom	Sums of squares	Mean square	Variance ratio	F probability
Cadmium	Concentrations	7	38,151.7	5,450.2	36.19	<0.001
	Residual	24	3,614.9	150.6		
	Total	31	41,766.6			
Copper	Concentrations	6	19,310.0	3,218.3	18.68	<0.001
	Residual	21	3,617.8	172.3		
	Total	27	22,927.8			
Iron	Concentrations	7	19,541.1	2,791.6	23.61	<0.001
	Residual	24	2,838.0	118.2		
	Total	31	22,379.1			
Zinc	Concentrations	7	13,469.5	1,924.2	8.94	<0.001
	Residual	24	5,166.1	215.3		
	Total	31	18,635.6			

## 3. Results and Discussion

The results from the mortality assay of *Artemia salina* nauplii at the stage of instar II-III were considered valid since the mortality percentage of the controls was 0%. The first experiment regarding the mobility assay was to record samples with ASW and 8 nauplii at time_0_ and time_24_. The results from these videos showed that the average speed of the nauplii at five different replicates remained at the same level for 24 h (Table 3). As a result, the movement of the nauplii is not affected by time in controls. This experiment was repeated for every experiment and always the results verified that the mobility of the nauplii remained constant after 24 h. Consequently, the movement of the nauplii was affected only from the existence of toxic compounds.

**Table 3:** Mobility of *Artemia salina* nauplii in ASW at time_0_ and time_24_.

Time (h)	Speed (m/s) per nauplius
0	0.011444
24	0.011636

The 24-h EC_50 _of potassium dichromate was estimated to 36.0 mg/L by the best fit model which is within the limits proposed by Svensson *et al*. [6].

The toxic effect of Cd^2+^ was obvious between 10^2^ mg/L where the nauplii retained the 100% of their mobility and 10^3^ mg/L where the nauplii had 25% of the initial mobility (Figure 1). The LC_50_ was estimated at 710.7 mg/L. The mobility of the nauplii, as evaluated by the camera-based instrument, had better sensitivity since the cadmium toxic effect was detectable at the concentration of 10 mg/L (100% mobility). The EC_50_ was 54.8 mg/L (Table 4), which is 13 times lower than the LC_50_ value. Kissa and co-workers [18] have estimated the 48 h LC_50_ at 159.6 mg/L. Furthermore, the experimental measurements were significantly different from the hypothetical values according to the t-test performed (p < 0.05).

The nauplii were not affected at the concentrations of 10^−4^–10^−1^ mg/L and retained 100% of their mobility according to the hypothetical curve in Cu^2+^ samples. Deterioration of the mobility was observed between 10^−4^ mg/L and 10^2^ mg/L, 84.1% and 10.9% mobility respectively by the camera-based device (Figure 1). The greater sensitivity allowed the detection of toxic levels even in very low concentrations. Furthermore, the EC_50_ value (7.6 mg/L) (Table 4) was 2.5 times lower than the LC_50_ (19.5 mg/L) and 1.3 times lower than the estimated 24 h LC_50_ of copper (9.5 mg/L) according to Gajbhiye and Hirota [4]. For this toxicant, the camera-based instrument proved more sensitive than the data in the literature and much more accurate than the mortality assay results.

The narrow range of 10^2^ mg/L and 10^3^ mg/L of Fe^2+^ proved toxic to *Artemia salina* nauplii according to the hypothetical curve. The LC_50_ could not be estimated due to extreme results. However, the lowest concentration detected as being toxic using the camera-based instrument was 10^−4^ mg/L Fe^2+^, which corresponds to 79.4% of their mobility (Figure 1). The EC_50_ was estimated at 66.3 mg/L (Table 4). The sensitivity range was extended 10^6^ times by the camera-based instrument comparing to the sensitivity range of the hypothetical curve. Although the 24-h EC_50_ was higher than the value of 18.2 mg/L determined by Gajbhiye and Hirota [4], the detection range was greater with the camera-based device. 

The hypothetical values achieved showed that the *Artemia salina* nauplii were sensitive to Zn^2+^ at 10^2^ mg/L and 10^3^ mg/L with 100% and 50% of their initial mobility respectively. By using the camera-based instrument higher sensitivity was achieved. The obtained curve presented a more constant trend with significantly different results from the hypothetical values according to the t-test performed (p < 0.05). The highest value of percentage mobility was observed at 10^−2^ mg/L corresponded to 88% mobility. The detection of toxic compounds proved successful at very low concentrations (Figure 1). The EC_50_ was 12.4 times lower (80.5 mg/L) than the LC_50_ value (1,000 mg/L) (Table 4). Gajbhiye and Hirota [4] evaluated the LC_50_ for zinc to 17.8 mg/L on *Artemia* nauplii. The high LC_50_ value might be due to the small population of shrimps used which lead to the increase of the mortal probability.

**Table 4:** The LC_50_ and EC_50_ values of the tested metals in mg/L as measured for the mortality assay (probit analysis) and camera-based instrument (best fit model).

Substance	LC_50_ (mg/L)	EC_50_ (mg/L) (±SD)
Cd^2+^	710.7	54.8 ± 11.1
Cu^2+^	19.5	7.6 ± 8.3
Fe^2+^	NE*	66.3 ± 9.7
Zn^2+^	1,000.0	80.5 ± 17.1

*NE: Not estimated.

Earlier studies on the toxic effect of heavy metals on the mortality of *Artemia* species have used very limited ranges in order the LC_50_ to be specified. For instance, Kissa *et al*. [18] have used a range of 1 to 200 mg/L. Gajbhiye and Hirota [4] have utilised different ranges dependent on the metal with a minimum of 0.1 mg/L and a maximum of 100 mg/L. Venkateswara *et al*. [19], who have studied the toxic effect of organophosphates on lethality of *Artemia salina*, also used a limited range of 0.2 mg/L to 3,000 mg/L dependent again on the tested toxic compound. In this study the EC_50_ values calculated with high sensitivity by using more extended scales. Moreover, *Artemia salina* species have been referred as organisms that accumulate toxic compounds with no effect on their life cycle. More specifically, it has been reported by Sarabia *et al*. [20] that *Artemia* species are very tolerant to cadmium exposure as mentioned before with LC_50_ values varied from 93.3 to 280 mg/L. On the contrary, this study has proved that the accumulation of toxic compounds has acute effects on the mobility of the nauplii after 24 h of exposure to the toxicants and this is measurable. This research was performed for quantifying the mobility of the nauplii in very low concentrations, which normally showed no affects in other studies. According to the results achieved this was successfully performed and the system showed high sensitivity detection levels extended to concentrations of parts per trillion. 

**Figure 3:**
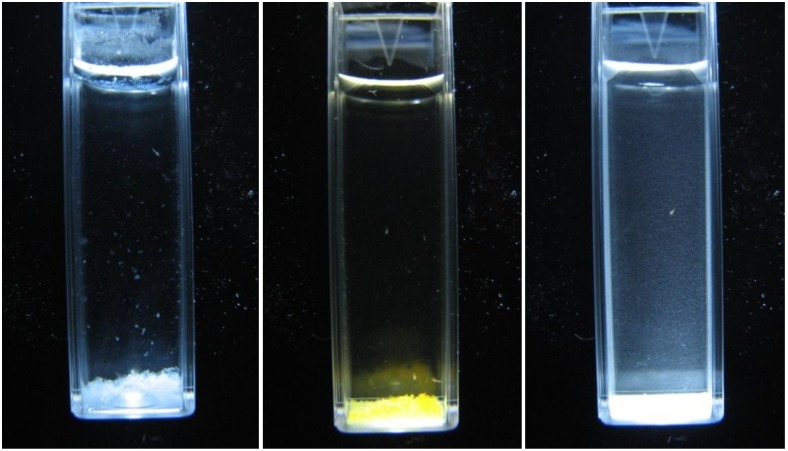
The first image shows the solution of 1,000 mg/L Zinc, the second one the iron solution of 1,000 mg/L and the third one Cadmium solution of 500 mg/L.

The software specifications have also minimised the possible errors such as the colour of the solution and the particulate settling to the minimum (Figure 3). 

Finally, the difference between the LC_50_ and EC_50_ values was noticeable in our experimental data. This happened due to the small populations used in the experiments. Using only four nauplii for assessing the mortality in toxic solutions showed very ambiguous results comparing to the results in the bibliography (Gajbhiye and Hirota [4]; Kissa *et al*. [18]) where the researchers used at least ten nauplii for each sample. In contrast, the mobility of the *Artemia salina* nauplii could be estimated with good sensitivity using the camera-based device even when the population is very low. Consequently, the calculation of the EC_50_ values even with small number of nauplii proved possible.

## 4. Conclusions

The results obtained demonstrate the ability of the system to accurately detect water toxicity in a short period of time and at low cost. The detection of toxic compounds was achieved in very early stages by estimating the mobility of the nauplii in toxic aqueous solutions and compared to the mobility of the organisms in control samples. The camera-based device can provide information about toxic compounds (including metals such as Cu, Cd, Fe and Zn) at a wide range of concentrations from 10^−4^ mg/L to 10^3^ mg/L. As a toxicity assay this approach is significantly more sensitive than the mortality-based assays, which can only detect concentrations starting from 10^−1^ mg/L. Furthermore, this study has proved that the accumulation of toxic compounds to *Artemia salina* nauplii has a deteriorating effect on their mobility. This deterioration was measured with high sensitivity by tracking the nauplii and estimating their average speed using digital image processing. Possible errors of the system achieved to be minimized and more accurate results to be obtained. The most of the findings achieved in this study have been verified from the literature and this leads to a reliable system with high sensitivity in low cost. Thus, this novel approach constitutes a more affordable instrument for promptly detecting toxic substances in aquatic solutions.
